# Spatial distribution and characteristics of injecting drug users (IDU) in five Northeastern states of India

**DOI:** 10.1186/1471-2458-11-64

**Published:** 2011-01-31

**Authors:** Gajendra Kumar Medhi, Jagadish Mahanta, Rajatashuvra Adhikary, Brogen S Akoijam, Buno Liegise, Kalpana Sarathy, Chelliah Joshua Thomas, Bhupen Sarmah

**Affiliations:** 1 Regional Medical Research Centre, NE Region, Indian Council of Medical Research, Dibrugarh-786001, Assam, India; 2 Family Health International (FHI), 16 Sunder Nagar, New Delhi 110003, India; 3 Community Medicine Department, Regional Institute of medical science, Lamphelpat, Imphal- 795004, Manipur, India; 4 Department of Education, Nagaland University, Kohima- 797 001, Nagaland, India; 5 Department of Social Work, Mizoram University, Aizwal-796006, Mizoram, India; 6 Indian Council of Social Science Research (ICSSR) North Eastern Regional Centre NEHU Campus, Shillong- 793 022, Meghalaya, India; 7 Omeo Kumar Das Institute of Social Change and Development, Six Miles, Guwahati-36, Assam, India

## Abstract

**Background:**

Injecting drugs is the major driving force of human immunodeficiency virus (HIV) epidemic in Northeastern India. We have assessed the spatial distribution of locations where injecting drug users (IDU) congregate, as well as the risk behaviour and key characteristics of IDUs to develop new strategies strengthening intervention measures for HIV prevention in this region.

**Methods:**

Locations of IDUs congregation for buying and injecting drugs were identified through Key Informants (KI). Verification of the location and its characteristics were confirmed through field visits. We also conducted semi-structured and structured interviews with IDUs to learn more about their injecting behaviour and other characteristics.

**Results:**

Altogether, 2462 IDU locations were identified in 5 states. The number of IDU locations was found to be greater in the states bordering Myanmar. Private houses, parks, abandoned buildings, pharmacies, graveyards, and isolated places were the most frequently chosen place for injecting drugs. Many injecting locations were visited by IDUs of varying ages, of which about 10-20% of locations were for females. In some locations, female IDUs were also involved in sex work. Sharing of needle and syringes was reported in all the states by large proportion of IDUs, mainly with close friends. However, even sharing with strangers was not uncommon. Needle and syringes were mainly procured from pharmacies, drug peddlers and friends. Lack of access to free sterile needles and syringes, and inconsistent supplies from intervention programs, were often given as the cause of sharing or re-use of needles and syringes by IDUs. Most of the IDUs described a negative attitude of the community towards them.

**Conclusion:**

We highlight the injection of drugs as a problem in 5 Northeastern India states where this is the major driving force of an HIV epidemic. Also highlighted are the large numbers of females that are unrecognized as IDUs and the association between drug use and sex work. Understanding of risk behaviours and other key charecteristics of IDUs in the region will help in strengthening harm reduction efforts that can prevent HIV transmission.

## Background

Abuse in injecting drugs and its association with human immunodeficiency virus (HIV) infection has become an important public health concern during last two decades in many countries worldwide. Some serious blood borne viral infections, e.g. hepatitis B (HBV) and hepatitis C (HCV) viruses adds to the misery. In India, illicit injection of drugs is already recognized as a major public health problem in Northeastern states. Heroin injection among IDU became popular in 1980s among the youths of the Northeast, particularly in Manipur [1]. The proximity of the Northeast to the Golden Triangle, the world's major point of illicit drug production, has traditionally been viewed as the most important reason for the increase.

Perhaps due to large scale sharing of needle syringes, seroprevalence of HIV among IDUs reached 50% by 1990 in Manipur [2,3]. Within a short span, 2 other northeastern states of India bordering Myanmar (Nagaland and Mizoram) also experienced a similar epidemic. The estimated prevalence of HIV among IDUs was 13% in Nagaland in 1998, and reached almost 10% in Mizoram by 2000 [4]. Prevalence also exceeded the 5% level in Assam in 2002 [5]. Subsequently, the epidemic spread to the wider populations from injecting drug users (IDU) through their sexual partners [6,7]. Manipur and Nagaland are 2 of the 6 HIV high prevalence states of India, where HIV among antenatal mothers is >1% [5]. Different strategies have been adopted to prevent HIV transmission among injecting drug users under the aegis of National AIDS Control Organization (NACO) since 1990s, including the supply of sterile injecting equipments and condoms; awareness and education programs on HIV prevention; clinical services for treating sexually transmitted infections (STI); referral to the voluntary counselling and testing Centre (VCTC); and peer-led outreach programs. A steady decline of HIV prevalence among IDUs in Manipur, Nagaland and Mizoram, possibly due to harm reduction efforts has occurred in recent times. Although declining, Manipur in 2007 still reported ~18% prevalence of HIV among the drug injecting population [8]. According to the last published report of the HIV sentinel surveillance in India, HIV prevalence in Nagaland, Mizoram, Meghalaya and Assam among IDUs were 2, 7.5, 4.2 and 2.2%, respectively [8]. However, recent reports suggest that, although HIV is declining, nevertheless other blood-borne pathogens, e.g. HCV, are creating even bigger problems in this region [9-11]. One recent report in 2009 indicated a 71% prevalence of HCV among IDUs in Mizoram [11]. Similarly, up to 78% prevalence of HCV among IDUs was reported from Manipur in 2008 [9]. Despite intervention programs in this region, there remain longstanding epidemics of HIV and other blood-borne pathogen problems among IDU populations. Further assessment is needed to find out the reasons for this and the spread of these problems to new areas.

Better understanding of the geographical extent of the drug injecting problem, key characteristics of the IDU population, their risk behavior, and societal attitude towards IDUs, is expected to help strengthen HIV prevention interventions in the region. Therefore, the study was conducted in 5 Northeastern states of India to: 1) map and assess the characteristics of locations where the IDUs could be found; 2) explore and describe the key characteristics of IDUs and their injection related risk behaviour, along with and their perception about attitude of community and healthcare professionals (HCP) towards them.

## Methods

### Settings

**Figure 1:**
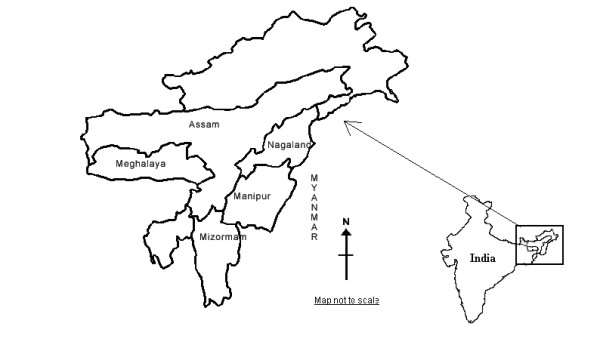
**Map of northeastern region of India indicating study states**.

The study was carried out in 2004. The Northeastern region of India consists of 7 states that include Arunachal Pradesh, Assam, Nagaland, Manipur, Mizoram, Tripura and Meghalaya. For operational reasons, only 5 states (Assam, Nagaland, Manipur, Mizoram, and Meghalaya; see Figure 1) were chosen. Three northeastern states (Manipur, Nagaland and Mizoram) share a common international border with Myanmar and have close proximity to the infamous golden triangle, the hub of the heroin trade. Except some parts of Manipur and Assam, the topography of the region is characterized by a hilly terrain. Assam is the most populous state of the region, with 26.6 million people. The population of other 4 states ranges from 0.9 to 2.4 million at the last population census in 2001.

### Design

Mapping was done to identify the locations where 2 or more IDUs congregated for injection of drugs or other purposes (purchasing, injecting and staying). Since 2 IDUs are the minimum requirement for sharing of injecting equipment, we considered a minimum threshold of two IDUs in defining an IDU location. An IDU was defined as a person of either sex, aged 18 years or older, who had injected drugs for non-medical reasons at least once in the last 6 months. Mapping process was facilitated by local KIs (Key Informants) who were knowledgeable about current and previous IDUs. To assess the characteristics of mapped IDU locations, multiple qualitative data collection methods were used, including observation, interviews with KIs and IDUs from the location. This data helped in cross-checking the accuracy of collected data and synthesizing the desired information. We also conducted structured and semi-structured interviews with IDUs to understand their injecting practices and other key characteristics. Separate study teams were formed for each state under the leadership of a state coordinator (BSA for Manipur, BL for Nagaland, KSD for Mizoram, CJT for Meghalaya and BS for Assam) to implement the study in their respective states. Before the mapping exercise, the study teams - led by the state coordinators - interviewed KIs from stakeholders associated with the drug using population in each state. The KIs were selected based on their ability to provide vital information on IDUs for the state. Initial interviews with KIs from major stakeholders provided preliminary insight about the geographical extent of the problem, and the probable locations where IDUs could be accessed. KIs also helped in selecting the district and sub-district level personnel who could facilitate mapping exercise, help in developing study tools, and plan our field work. They were included from the State AIDS Control Societies (SACS; 2 from each state), non-governmental organizations (NGOs)/community-based organizations (CBOs) working with IDUs (3-9 from each state), drug de-addiction centres (DDC; 2-5 from each state), narcotic/excise departments (2 from each state), police departments (2-3 from each state), health departments (2-3 from each state), pharmacies (2-3 in each state), people living with HIV/AIDS (PLWH) (1-3 from each state, but none from Meghalaya and Mizoram) and social welfare department (2 from each state). Altogether, 118 interviews were conducted (30 each in Manipur and Nagaland, 20 each in Mizoram and Meghalaya, and 18 in Assam).

### Data Collection

Mapping of the locations were carried out by the field teams through extensive field visits in all the 5 states, covering every district and sub-division. People acquainted with IDUs (e.g. outreach workers and other persons from NGOs associated with IDU intervention programs, current and ex-IDUs) acted as KIs to facilitate contact with IDUs.

The snowballing technique (chain of referrals) was used to identify the locations. The initial KIs were asked to provide further information about other people able to provide more information about places of IDUs congregation. This process of identifying KIs continued until no more new information was forthcoming. Similarly, IDUs in one location were also requested to act as KIs for further identification of IDU locations. This process continued until no new information about IDU locations was forthcoming. The KIs were requested to lead the study teams to the locations during peak time of congregation. Visiting during the peak time helped in better assessment of the characteristics and activities of the study population. To ensure quality data, >10% of locations identified by the field teams were cross-verified by the state coordinators and other investigators. The research teams involved in the execution of the study were trained by social scientists on various methods and techniques to be adopted for collecting data. After identifying the locations, the field teams prepared a hand-drawn sketch of the identified location with its key landmarks, boundaries and other features. The field teams spent some time in the locations interacting with the drug users to build up a rapport with them. The current/ex-IDUs in the team introduced the field teams to IDUs in the locations and helped in building trusting relationship between the field team members and IDUs. The field teams in this process assessed the characteristics of identified locations through direct observations, interaction with IDUs and the KIs. All the information during mapping process was collected using a format developed for this purpose. The data included a geographical description of the identified location (e.g. state, district, urban/rural, name of the village/town, important landmark by which the location could be identified), types of locations (physical characteristics of locations), the nature of the location (drug and sex work site, shooting gallery), demographic information of IDUs (age and gender), and average numbers of IDUs congregated in the locations per day. Based on information obtained during preliminary exploratory interviews with KIs, types of locations were pre-coded into 8 categories (such as home, pharmacy, parks, abandoned building, cemeteries/graveyards, riverside, public toilet, and tea stalls). However, any new locations found during the survey were also noted in the format apart from these 8 categories. The average number of IDUs usually assembled in each location was determined by asking the KIs or IDUs available in the locations. Similarly, information about the age ranges of IDUs assembled in each location was also determined by asking the KIs or IDUs. A shooting gallery was defined as a place where injecting equipments were readily available, where it could be purchased, borrowed or rented. A drug and sex work site was defined as an IDU location where IDUs had sex within the group.

Semi-structured qualitative interviews with IDUs were also conducted to learn about participants' needle/syringe sharing behaviour and sharing relationships, network size, sources of acquisition of sterile needles and syringes, and their perception about attitudes of community and healthcare providers (HCP) towards them. Additionally, structured closed-ended questions were also asked to elicit some key information (viz. needle/syringe sharing behaviour in the past 6 months and network size). IDUs present in the location during the visit of the study team were recruited purposively for the interviews. Interviews took ~15 minutes. Only IDUs aged 18 years or over who had injected drug at least once in the last 6 months were interviewed. Participants who were under the influence of drug (intoxicated) and aggressive were excluded from interviews. Except for Nagaland (where 2-3 IDU were interviewed), at least one IDU was interviewed from each selected location. To assess the network size, aggregate network data [12] was collected from IDUs by asking "How many IDUs do you know personally, and in turn who also knew you?" To assess their needle/syringe sharing practices, IDUs were asked if they had injected with needle/syringes previously used by other IDUs, or passed on their used needle/syringes to others in the last 6 months.

### Ethical consideration

The study protocol was reviewed and recommended for implementation by the Institutional Ethical Committee of Regional Medical Research Centre (RMRC) of Indian Council of Medical Research (ICMR). All the interviews were unlinked and anonymous. No names or any other personal identifiers of the participants were recorded anywhere. No information on the target group recruited was shared with any outside groups. The data regarding identified locations were not shared with any outside parties. No interview commenced without prior oral consent of the respondents. The informed consent form was either read aloud or thoroughly explained to the respondents, and once the respondent had provided oral consent, a member of the teams signed the consent form on behalf of the respondent.

### Data Analysis

The locations of IDUs were categorized according to various characteristics identified during mapping exercise. All the semi-structured interviews were tape-recorded and transcribed verbatim. The transcripts were cross-checked again with the interview tapes. Transcriptions were coded manually to identify important, recurrent themes and categories pertaining to the questions of interest. All the transcripts were read carefully by 2 independent teams to extract the themes and categories. Initial codes were developed jointly by the investigators to identify the themes and categories based on detailed reviews of 4/5 transcripts from each state. However, codes were further refined as new themes or categories emerged from the data during the coding process. Any disagreement between two coding teams was resolved by discussion. Results of closed-ended-structured questions (e.g. needle/syringe sharing behaviour in the past 6 months and network size) were expressed as a percentage of the total responses. Analyses were done separately for each state.

## Results

### Mapping of locations

A total of 2462 IDU locations were identified, with the states sharing a common border with Myamar, i.e. Manipur, Nagaland and Mizoram, having the highest number of locations. Manipur had the highest umbers of sites (1337 locations) followed by Nagaland (475 locations), Mizoram (294 locations), Meghalaya (186 locations), and in Assam 170 locations were found. The number of locations identified per thousand km^2 ^was highest in Manipur, followed by Nagaland and Mizoram. Assam had the lowest numbers of IDU locations per thousand km^2 ^(Table 1). The characteristics of IDU locations assessed during mapping process are described state-wise follow:

**Table 1:** Distribution of states according to total populations, land areas, numbers of locations

Sl No	States	Population (million)	Land area (sq km)	Numbers of locations
1	Manipur	2.17	22,327	1337
2	Nagaland	1.99	16,579	475
3	Mizoram	0.89	21,087	294
4	Meghalaya	2.39	22,429	186
5	Assam	26.66	78,438	170

### Manipur

Of the 1337 IDU locations in Manipur, ~76% were in rural and 24% in urban areas. Private residences were the most common gathering places of IDUs (45%) (Table 2). Other important locations identified were secluded places such as riversides (8.8%), graveyards (5.2%), abandoned buildings (2.3%), public parks (3.4%), public toilets (1.3%), etc. Four IDU locations were also identified as sex work site. Location-wise average numbers of IDUs (average daily congregation in the locations) are given in Table 3. On average, 6-10 IDUs assembled in ~48% of the locations daily, followed by 11-20 IDU in ~26% locations. More than 20 IDUs also assembled in a large number of locations. IDUs were mostly between early twenties and mid thirties. Although the majority of locations (51%) were used by IDUs of different ages (≤25 years and >25 years), 20% were mostly used by IDUs aged 25 years or less and ~29% by those aged over 25 years. No exclusively female IDU locations were identified. However, there were female IDUs in ~10% of the identified locations.

**Table 2:** State-wise types of locations of IDUs

	States				
	
Types of Sites	Manipur n (%)	Nagaland n (%)	Mizoram n (%)	Meghalaya n (%)	Assam n (%)
Home	602 (45)	302 (63.6)	49 (16.7)	66 (35.5)	18 (10.6)
Riverside	117 (8.8)	10 (2.1)	35 (11.9)	2 (1.1)	8 (4.7)
Parks	45 (3.4)	4 (0.8)	13 (4.4)	4 (2.1)	8 (4.7)
Graveyards	69 (5.2)	3 (0.6)	38 (12.9)	10 (5.3)	7 (4.1)
Pharmacy	3 (0.2)	14 (2.9)			22 (12.9)
Abandoned building	31 (2.3)	54 (11.4)	22 (7.5)	3 (1.6)	4 (2.4)
Public Toilet	17 (1.3)	21 (4.4)	3 (1)	3 (1.6)	14 (8.2)
Tea Stalls	22 (1.6)	15 (3.2)	1 (0.3)	21 (11.2)	14 (8.2)
Others	419 (31.3)	52 (10.9)	133 (45.2)	79 (42.5)	75 (44.1)

**Table 3:** State-wise distribution of IDU locations according average numbers of IDUs

	States				
	
Numbers of IDUs per locations	Manipur ^(%)^	Nagaland ^(%)^	Mizoram ^(%)^	Meghalaya ^(%)^	Assam ^(%)^
≤5	225 (16.9)	109 (22.9)	81 (32.5)	56 (29.8)	19 (11.2)
6-10	645 (48.3)	183(38.5)	111 (44.6)	103 (54.7)	67 (39.4)
11-20	349 (26.1)	130 (27.4)	25(10)	25 (13.3)	54 (31.8)
>20	116 (8.7)	53 (11.2)	32 (12.9)	4 (2.1)	30 (17.6)

### Nagaland

Of the 475 locations identified in Nagaland, 72% were located in urban areas and 28% in rural areas. Similar to Manipur, private residences were the most common gathering places (63.6%) (Table 2). Other locations were abandoned buildings (11.4%), public toilets (4.4%), tea stalls (3.2%), pharmacies (2.9%), and river-side (2.1%). A few locations were also used as shooting galleries in the state. Six IDU locations were identified as being used for sex work. An average 6-10 IDUs congregated in ~39% of the locations daily, whereas ~23% of locations were used by <6 IDUs (Table 3). IDUs in 33% of the locations had mostly IDUs of ≤25 years. On the other hand, ~30% were used by IDUs over 25 years of age. However, a substantial number of locations (37%) was used by IDUs of different ages. IDUs were mostly male (80%). And no exclusively female IDU sites were identified.

### Mizoram

Of a total of 294 IDU locations in Mizoram, the majority (78%) were in urban areas. In contrast to Manipur and Nagaland, IDUs in Mizoram congregated more in outdoor/public places than private residences (~17% for the latter; Table 2). Other places were graveyards (12.9%), riverside (11.9%), and abandoned buildings (7.5%). Six shooting galleries were also identified and another 6 locations were also used for sex work. About 45% of the locations in Mizoram were used by 6-10 IDUs daily, whereas 33% locations were used by <6 IDUs (Table 3). About 40% of locations were used by IDUs of different ages (18-35 years) while 30% were used exclusively by relatively younger IDUs (≤25 years) and another 30% of locations by aged >25 years. About 82% of locations were exclusively accessed by male IDUs only, and rest was used by both male and female IDUs.

### Meghalaya

Of a total number of 186 IDU locations identified in Meghalaya, 81% were in urban areas. About 35% locations were identified in private residences (Table 2), but other important locations were various public places, such as tea-stalls, taxi-stands, liquor shops, market areas, bus-stops and playgrounds, or isolated places such as graveyards, forests, river-sides, abandoned-buildings. Thirteen sites were also used for sex work. In the majority of the locations (~55%), 6-10 IDUs used to congregate daily, whereas ~30% were accessed by <6 IDUs (Table 3). The majority of IDUs were between 18 and 25 years old in all the locations. Some of them were reported to be <18 years old. About 90% of locations were exclusively accessed by male IDUs, whereas the rest were accessed by IDUs of either sex.

### Assam

Although Assam was the most populous of the 5 states, the number of IDU locations identified was the fewest of all the states. Most of the locations (85%) were situated in urban areas. Unlike Manipur, Nagaland, Meghalaya, only a few locations were private residences (10.6%). Other places, such as pharmacies (n = 22), tea stalls (n = 14), public toilets (n = 14), parks, riverside, cemeteries, abandoned buildings, auditoriums, saloons, bazaar area, bus stands, farm houses, forest areas, godowns (store-rooms), railway stations, and places used for religious purposes, could be identified as injecting locations. Four locations were also found to be used for sex work. On average, 6-10 IDUs assembled in ~39% of the locations and about ~32% were frequented by 11-20 IDUs (Table 3**)**. About 18% of the locations were used by >20 IDUs daily. The IDUs assembled in most of the locations (69%) were of different ages (18 to 35 years). In some locations (24%), IDUs <18 years of age were encountered along with others. About 7% of exclusive teenager (<18 years) locations were also identified. About 90% of locations were exclusively used by male IDUs, with the rest being for either sex.

### Interviews with IDUs

#### Manipur

**Figure 2:**
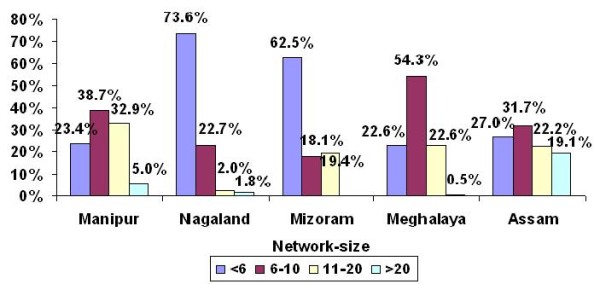
**Percentage distribution of IDUs according to self-reported aggregate network size**.

**Figure 3:**
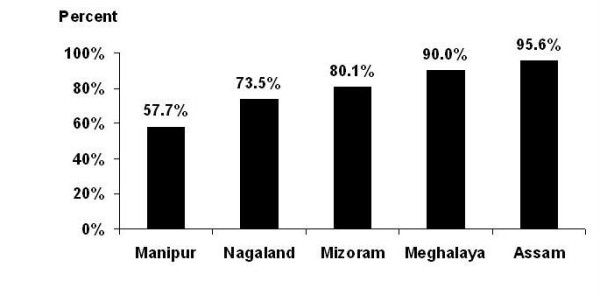
**Percentage of IDUs who shared needle/syringes in last six months**.

A total of 1337 male IDUs were interviewed in Manipur. The aggregate network size of 6-10 was reported by 39% of IDUs, 11-20 by 33% and >20 by 5% (Figure 2). The majority of the IDUs (58%) said they had shared needles and syringes during the past 6 months (Figure 3), but most of them shared only with their close friends and regular injecting partners. About one third of IDUs shared needle and syringes with IDUs other than their usual injecting partners. Some IDUs even admitted having shared with strangers. Despite the presence of NSEPs in all the districts, most of the IDUs acquired needles and syringes from non-NSEP sources, such as pharmacies, peddlers and friends on payment. Many of them reported sharing or re-using needles and syringes because they were unable to purchase syringes regularly from pharmacies or peddlers. A major concern was that they shared or re-used needles and syringes, often without proper sterilization. This was echoed in the following statement of an IDU:

"Syringes are available from the drug sellers and friends only (on payment). So, if we want to inject we have to re-use the discarded needles and syringes by washing with plain water. Often we inject from same needle-syringes in the location."

About 37% of respondents reported that they mainly procured sterile needles and syringes from existing programs (needle syringe exchange programs -NSEP) operated through drop-in-centres (DIC) or through outreach workers. But many of them also reported sharing or re-using needle and syringes on many occasions because of an interruption in the supply of sterile equipments from NSEPs. One IDU described the situation:

"I use to take syringes from DIC. I also often share needles and syringes with my friends because many times supplies of needles and syringes are stopped. I often clean needles and syringes with plain water or bleach before I share with my friends.*"*

Similarly, another IDU said:

*"*We get needles and syringes from outreach workers or from DIC. Sometimes, it is difficult get needles and syringes from outreach workers or DIC because of a sudden bandh, a general strike. During this time we used to clean old used syringes and re-use them or share syringes among ourselves."

Similar description was also given by another IDU:

"Our main source of acquiring needles and syringes is from NGOs. But as this is not available throughout the year, we start sharing among our friends. Also, it is not possible to get clean needles and syringes every time we need them, so we are compelled to share among ourselves."

Most of the IDUs described HCPs as cordial and non-discriminatory towards them. However, some IDUs felt differently. One IDU revealed his experiences with HCPs:

"I was suffering from diarrhea. When I went to a hospital for treatment, the doctor refused to treat me, knowing that I am a drug user. Then I went to a NGO working in the field of HIV/AIDS and took my treatment there."

The majority of the IDUs interviewed felt that the attitude of the general community towards them was largely negative and hostile. Many of them reported receiving threats or even physical punishments (e.g. beating) from local underground militant groups for being drug users. IDUs were highly stigmatized in the general community. The main reason for stigma attached to injecting drug use was its association with HIV/AIDS. An IDU stated:

*"*Society looks down upon IDUs because they think that IDUs spread HIV/STIs and other diseases."

#### Nagaland

A total of 988 male IDUs were interviewed in Nagaland. The majority of IDUs (74%) reported smaller network size (<6). About 23% of IDUs reported their network size to be 6-10 and a small proportion had 11-20 or >20 IDUs in their network (Figure 2). Close to 74% of IDUs reported having shared needles and syringes during past 6 months (Figure 3). Like Manipur, the majority of the IDUs said that they shared only with their close friends and with the same injecting partners. However, many IDUs reported to have shared needles and syringes with IDUs outside their usual group. Some IDUs also reported sharing with strangers and new IDUs. The majority of the IDUs indicated that pharmacies and peddlers were the only sources of obtaining fresh needles and syringes, as in Manipur. At the same time, they also reported that they were unable to purchase fresh needles and syringes from pharmacies or peddlers for every injection. Therefore, they most often engaged in syringe sharing with friends or the re-use of previously used needles and syringes. This situation was described by one IDU:

"I cannot afford to buy needles and syringes every times whenever I fix; hence I re-use my old needles and syringes by cleaning them with spirit or plain water."

Some desperate users sometimes picked up needles and syringes from sources such as hospital waste-bins to meet their needs. This was illustrated by an IDU:

"Many IDUs pick up needles and syringes from the waste-bins of hospitals, clients, pharmacies and even veterinary hospitals."

About 30% of the interviewed IDUs reported that they usually acquired needles and syringes from available interventional programs. But, like Manipur, many of them who acquired needles and syringes from NSEPs sometimes resorted to risky injecting practices, such as sharing or re-using syringes because of an interruption in the supply from NSEPs.

In general, IDUs consistently reported that HCPs were cordial or friendly towards IDUs. However, some of them said they faced discrimination from doctors and other HCPs. According to majority of the participants, the attitude of the community towards IDUs was largely negative. Most of the IDUs felt that they were discriminated against, marginalized, looked down upon, and rejected by the society. On many occasions, IDUs were beaten up and even locked up for using illicit drugs. One IDU said:

"The community is less concerned about IDUs. We are hated by the community members and church overlooks us. We are isolated from social gatherings and are considered sinners and criminals".

#### Mizoram

A total of 225 IDUs (including one female IDU) were interviewed. The majority of IDUs (63%) reported a smaller network size (<6 IDUs) (Figure 2). About 80% of IDUs reported having shared needles and syringes with others in the past 6 months (Figure 3). It was common to share needles and syringes with IDUs who were not their usual injecting partners. They also reported sharing with IDUs from other localities; sharing with new injectors and strangers was not uncommon. However, a large proportion of them also reported sharing only with their close friends and their regular injecting partners. The majority of IDUs had to purchase or borrow needles and syringes from pharmacies, peddlers and friends. Only a few interviewed IDUs reported that they had access to free sterile needles and syringes from intervention programs. Therefore, despite being aware of the adverse consequences, they often engaged in risky injecting practices, e.g. re-using or sharing of needles and syringes.

Most of the IDUs said that they were well behaved by HCPs and not discriminated against because of their drug habit. However, a small number of them felt discriminated against by HCPs for being IDUs. The community at large considered drug use a "bad practice" and a "social evil". But, according to most of the IDUs, the attitude of the community was mainly positive towards them. Some of them said that they were not subjected to social rejection and isolation, but, many IDUs perceived the community's attitude towards them as negative and quite discriminatory.

#### Meghalaya

A total of 192 IDUs (182 male, 10 female) were interviewed. About 54% reported 6-10 IDUs in their network (Figure 2), and over a fifth (23%) of them had 11-20 IDUs in their network. Sharing of needles and syringes appeared to be very high in Meghalaya as >90% of interviewed IDUs admitted having shared needles and syringes in the past 6 months (Figure 3), and most had shared with other IDUs who were not their usual injecting partners. Some of them also mentioned that they had sometimes also shared with complete strangers. However, many of them also said that they shared needles and syringes only with their regular injecting partners who were usually their close friends. One IDU said:

"I share only with my group members and usually clean needles and syringes with plain water."

The overwhelming majority of interviewed IDUs reported a lack of access to sterile needles and syringes from NSEPs. They had to purchase or borrow injecting equipment from pharmacies, friends or peddlers who contributed in needle syringe sharing and re-use among IDUs.

The experiences of IDUs with HCPs were mainly positive. Community at large considered the IDUs as useless, a menace and burden to the society. According to some IDUs, they were often tortured and threatened to give up the drug habit. Most of the respondents felt that they were discriminated against, marginalized and stigmatized by the society. IDUs also tended to hide their injecting behaviour to avoid negative consequences. One IDU said-"Nobody knows except my close friends that I use drugs."

#### Assam

In Assam, a total of 111 male IDUs were interviewed. About a quarter of IDUs (27%) reported a smaller network size (<6 IDUs), while approximately one fifth (19.1%) of them reported >20 IDUs in their network (Figure 2). Most of the IDUs (96%) admitted that they had shared needles and syringes in the past 6 months with other IDUs. Sharing with unknown partners was reported by many IDUs. Sharing with IDUs from other places, even from neighboring states (Nagaland and Manipur) was reported by some IDUs. Like other states, sharing or re-use of syringes occurred mainly because of the lack of access to free sterile needles and syringes. Needles and syringes were mainly purchased from pharmacies, but some of the IDUs also purchased or borrowed them from drug peddlers and friends.

Most of the IDUs said that they were treated well by the HCPs. Many IDUs perceived doctors as "supportive", "sympathetic" and "friendly" towards them. Most of the IDUs felt that the attitude of general community was negative and discriminatory towards IDUs; they were considered anti-social and a burden to society.

## Discussion

The Northeastern region is experiencing the highest IDU-related HIV transmission in India and this study has revealed the extent of the injecting drug use problem as well as some of the key characteristics of IDUs in 5 states. In contrast to the popular belief that the injecting drug use problem was mainly concentrated in urban areas, this study identified many rural locations of IDUs. In the worst hit state, Manipur, >70% of sites were identified in rural and remote areas. Therefore, the challenge of harm-reduction programs is to reach IDUs in these locations with difficult geographical terrains overcoming the socio-environmental barriers [9,13]. As expected, the study demonstrates that the injecting drug use problem was more extensive in 3 of the states (Manipur, Nagaland and Mizoram) bordering Myanmar. National Aids Control Organization (2006) of India estimated that Manipur was the worst hit state with IDU problem, followed by Nagaland in the whole country. Mizoram had the third highest numbers of IDUs among northeastern states of India [5].

Majority of the IDU locations were residential homes in the states of Manipur and Nagaland. Subsequent studies carried out in these states reconfirmed our findings [9]. In such settings, identification and providing services to IDUs presents a major challenge [9]. Homes may be safer place for IDUs because of the social unacceptability and clandestine nature of drug use. However, in Assam and Meghalaya, injecting drug use is still sporadic and IDUs mostly inject outdoors and in isolated places, such as graveyards. People usually avoid frequent visits to these places and perhaps for this reason they were chosen for such clandestine activities. The presence of shooting galleries in some states is of great concern because these are considered not only high risk environment for individual users, but may also act as repositories of contaminating pathogens. Use of contaminated equipment spread pathogens from an infected individual to uninfected users [14,15].

Earlier studies showed that younger IDUs who injected with older subgroups or with those who have injected for longer duration were at increased risk of blood-borne infection because of the higher prevalence among the older subgroups [15-17]. In many sites across the states, younger and new users were found to be injecting with older IDUs, making themselves more vulnerable to the blood-borne infections from older sub-groups. Data regarding the duration of drug use was not collected in this study. Nevertheless, findings of a cross-sectional study involving several districts in this region suggest that older IDUs usually have longer drug using "careers" [9]. In Manipur, prevalence of HIV among the IDU population is ~20% and about three quarter of IDUs is also infected with HCV [8,9,11]. Hence, in such settings, new IDUs are certainly at greater risk of contracting blood-borne infections from older IDUs through sharing injection equipment. Interventional efforts should therefore focus more on young IDUs before they get infected with blood-borne infectious agents [9].

Injecting drug use is perceived predominantly as a male problem in India. However, this study highlighted significant presence of female IDUs in all the 5 northeastern states. Further, evidence of sex work in some locations added a new facet in the transmission dynamics of HIV infection. Female IDUs may often sell sex for drugs and for their livelihood in the region [19]. It appears that the dual risks of sexual and drug injecting practices put female IDUs in this region at greater risk of the acquisition and transmission of HIV infections. An earlier study in Manipur reported a high prevalence of HIV (57%) among female IDUs [18]. Female IDUs also acting as sex workers in the region may facilitate transmission of HIV infections to a wider general population through their non-IDU sexual partners.

Sizes of injecting networks, and their density and connectivity are key determinants of transmission of blood-borne infection among IDUs [19-25]. The larger size of injecting network may have contributed to the rapid spread of HIV in some parts of Pakistan [20]. Moreover, a higher level of personal network density and larger drug network size are positively associated with needle sharing [25]. During interviews in the present study, many IDUs disclosed having bigger personal network (11-20 or more). Many of them also reported sharing needles and syringes with non-regular injecting partners and strangers. Recent studies have reconfirmed that the IDU population in Manipur, Nagaland and Mizoram have higher levels of shared injecting equipment use [9,11]. In the present study, many IDUs reported obtaining their needles and syringes from sources other than government or non-government organization run NSEPs, which might have contributed to sharing. In the worst hit states, Manipur and Nagaland, obtaining needles and syringes from NSEPs was lower despite the existence of interventional programs. The pharmacy was an important source of acquiring needles and syringes among IDUs. However, our findings suggest that the inability to afford to purchase sterile syringes regularly forced many IDUs to share or re-use syringes. Also, in a setting where drug use is not a socially acceptable behaviour, IDUs may not like openly to purchase syringes from pharmacies. Although pharmacies can be added sources of sterile needles and syringes, and provide benefit in addition to those derived from NSEP [26], cost and visibility often prevent many IDUs from procuring needles and syringes from pharmacies in this region.

Continued risky injecting practices among IDUs, despite the presence of NSEPs, may be attributable to a number of factors, including environmental barriers. The socio-political environment in states such as Manipur and Nagaland affects the consistency of services and safety of NGO staffs and IDUs [9,13]. Program workers may often fail to reach IDUs to distribute sterile injecting equipments for safe drug injection due to disturbing situations. On many occasions, distribution of sterile injecting equipment to IDUs is also hampered by regular police harassment of outreach workers if they were found with needles and syringes [9,13,27]. Many IDUs in our study also revealed that the IDUs did not have any other alternative than re-using old needles and syringes or sharing them with other IDUs due to the inconsistencies of interventional programs.

The discriminatory and negative attitude of the community in general towards IDUs is also a major barrier in implementing interventions in this region [13]. Therefore, to reduce the negative attitudes of the community towards IDUs, there needs to be an enhancement of community mobilization effort. Environmental barriers encountered in implementing outreach programs can be minimized through mobilizing the community at large before targeting the IDU population [13]. However, more research is needed to understand the causes of continued high-risk injecting behaviour of IDUs in the region [9,11].

This study has certain limitations that need to be mentioned. Despite our best efforts, we could not cover some remote difficult-to-reach areas in the states. Hence, the problem in the rural and remote areas may be bigger than we have actually found in this study. Secondly, many IDUs may not often congregate at identifiable and accessible locations because of the clandestine nature of IDUs' behaviour. Thus many groups in hard-to-reach locations remained relatively unexplored. Therefore, the findings of the assessment may not reflect the true magnitude of the injecting drug use problem in different states. However, the findings of this assessment may indicate adequately the severity of the problem in different states that is consistent with other assessments [5]. Thirdly, recruitment of IDUs for the interview was purposive; hence the findings of the study may be affected by the bias inherent in purposive sampling. Fourthly, except for Meghalaya and Mizoram, no female IDUs were interviewed in the other 3 states. Therefore, findings of structured and semi-structured interviews may not be generalized to all female IDUs.

## Conclusions

The study has provided useful information on the injecting drug use problem in the Northeastern region of India where injecting drug use is the main driver of the HIV epidemic. The considerable presence of IDUs in the remote and rural areas of the region with difficult terrain underscores the need to expand the harm reduction programs to rural areas. The study has also demonstrated that risky injecting practices, such as the sharing or re-use of needles and syringes, is common among IDUs in all the states. Needles and syringes were mainly procured from non-NSEP sources by IDUs, which contributed to the sharing of needles and syringes. Therefore, there is urgent need to scale up NSEP programs to ensure uninterrupted access to sterile injecting equipment to prevent risky injecting practices among IDUs. Mapping of IDU sites will be useful in designing strategies for reaching them. The study also highlighted the largely unexplored injecting drug use problem of females in this region. A more innovative strategy will be required to reach this relatively inaccessible sub-group. Sharing of the same location by younger and older IDUs, drug and sex interfaces are some important findings of the study.

## Abbreviations

CBO: Community based organization; DDC: Drug De-addiction Centres; DIC: Drop-in-centre; HIV: Human Immunodeficiency Virus; HBV: Hepatitis B Virus; HCV: Hepatitis C Virus; HCP: Health Care Providers; IBBA: Integrated Biological and Behavioural Assessment; ICMR: Indian Council of Medical Research; IDU: Injecting Drug User; KI: Key Informants; NACO: National AIDS Control Organization; NGO: Non-governmental Organization; NSEP: Needle and Syringe Exchange Program; PLWH: People Living with HIV/AIDS; RMRC: Regional Medical Research Centre; STI: Sexually Transmitted Infections; SACS: State AIDS Control Society; VCTC: Voluntary Counseling and Testing Centre.

## Competing interests

The authors declare that they have no competing interests.

## Authors' contributions

JM and RA conceived and designed the study; GKM contributed in study design, oversaw the data collection, led data analysis, drafted the manuscript and incorporated suggestions form other authors. JM reviewed the manuscript. All the co-authors (BSA, BL, KD, CJT and BS) contributed in data collection, interpretation of the data and drafting of the manuscript, and all the authors approved the final version of the manuscript.

## Funding Agency

Support for this study was provided by Family Health International (FHI) with funds from United States Agency for International Development (USAID) award number HRN-A-00-97-00017-00), although the views expressed in this article do not necessarily reflects those of FHI or USAIDS.

## Pre-publication history

The pre-publication history for this paper can be accessed here:

http://www.biomedcentral.com/1471-2458/11/64/prepub
